# Supplementary figures and images for: PhenoTrack3D: an automatic high-throughput phenotyping pipeline to track maize organs over time
Source: Plant Methods. 2022 Dec 8;18:130. doi: 10.1186/s13007-022-00961-4 (PMC9730636; doi:10.1186/s13007-022-00961-4)

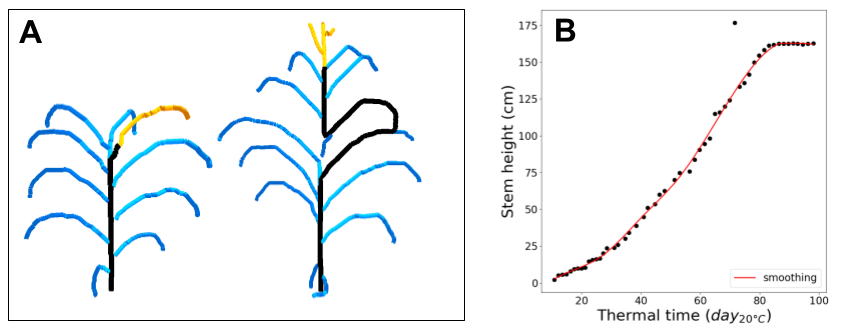

Supplement: Supplementary file 3 — Additional file 3. Example of the anomaly detection step for one maize plant. A) two reconstructed plants removed during anomaly detection due to their abnormal stem shapes. B) Stem height smoothing over time, allowing to correct an outlier. [file 13007_2022_961_MOESM3_ESM.png]

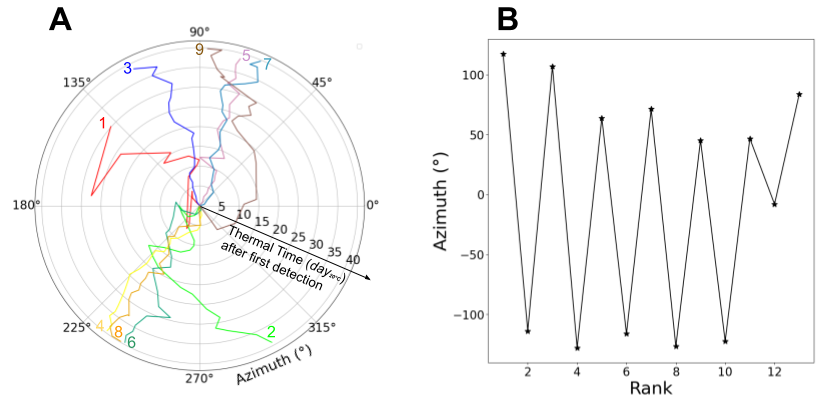

Supplement: Supplementary file 4 — Additional file 4. Example of azimuth traits extracted with the pipeline for one maize plant. A) Azimuth dynamics of individual leaves, up to 40 day20°C after their first detection. B) Leaf azimuth profile. [file 13007_2022_961_MOESM4_ESM.png]

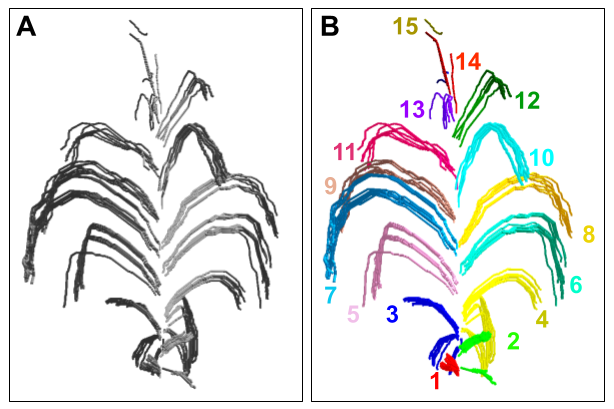

Supplement: Supplementary file 5 — Additional file 5. Visualisation of rank assignment following sequence alignment on a set of 3D ligulated leaf polylines. A) Visualisation of all ligulated leaves polylines in a time-series of 3D reconstructions of one plant. B) Assignment of leaf ranks on this set of polylines, using sequence alignment. [file 13007_2022_961_MOESM5_ESM.png]
